# A versatile polyacrylamide gel electrophoresis based sulfotransferase assay

**DOI:** 10.1186/1472-6750-10-11

**Published:** 2010-02-10

**Authors:** Zhengliang L Wu, Cheryl M Ethen, Sara Larson, Brittany Prather, Weiping Jiang

**Affiliations:** 1 R&D Systems Inc, 614 McKinley Place NE, Minneapolis, MN 55413, USA

## Abstract

**Background:**

Sulfotransferases are a large group of enzymes that regulate the biological activity or availability of a wide spectrum of substrates through sulfation with the sulfur donor 3'-phosphoadenosine-5'-phosphosulfate (PAPS). These enzymes are known to be difficult to assay. A convenient assay is needed in order to better understand these enzymes.

**Results:**

A universal sulfotransferase assay method based on sodium dodecyl sulfate polyacrylamide gel electrophoresis (SDS-PAGE) is described. This assay has been successfully applied to substrates as small as α-naphthol and as big as proteoglycans. As examples, we present the assays for recombinant human CHST4, TPST1, CHST3 and HS6ST1. In order to assess whether a small molecule can be applicable to this type of assay, a method to estimate the relative mobility of a molecule to PAPS is also presented. The estimated relative mobilities of various sulfated small molecules generated by SULT1A1, SULT1E1, SULT2A1 and CHST4 are in the range of ± 0.2 of the actual relative mobilities.

**Conclusion:**

The versatility of the current method comes from the ability that SDS-PAGE can separate proteins and small molecules according to different parameters. While mobilities of proteins during SDS-PAGE are inversely related to their sizes, mobilities of small molecules are positively related to their charge/mass ratios. The predicted relative mobility of a product to PAPS is a good indicator of whether a sulfotransferase can be assayed with SDS-PAGE. Because phosphorylation is most similar to sulfation in chemistry, the method is likely to be applicable to kinases as well.

## Background

Sulfation is a ubiquitous post-translational modification that affects the biological activity of a wide variety of substrates, ranging in molecular mass from less than 10^3 ^to greater than 10^6 ^Da. The reaction is catalyzed by sulfotransferases using 3'-phosphoadenosine-5'-phosphosulfate (PAPS)^1 ^as the sulfate donor [1]. There are 48 sulfotransferases in humans and they can be divided into two major groups according to their sub-cellular localization: those found in cytoplasm and those found in the Golgi apparatus [2]. Cytosolic sulfotransferases, commonly designated as SULTs, are mainly involved in modifying small molecules (in general < 1000 Da in mass), such as steroids, neurotransmitters, and xenobiotics [3]. Along with P450 enzymes, SULTs play important roles in drug detoxification [4,5]. Golgi resident sulfotransferases are involved in modifying numerous glycans and proteins on the cell membranes and within extracellular matrix. Glycan sulfation is known to affect hormone pharmacokinetics [6], growth factor and cytokine activity [7], viral [8] and bacterial [9] invasion. Protein sulfation is known to be important in protein-protein interactions, such as leukocyte adhesion molecule PSGL-1 binding to P-selectin on activated endothelium [10,11].

**Figure 1:**
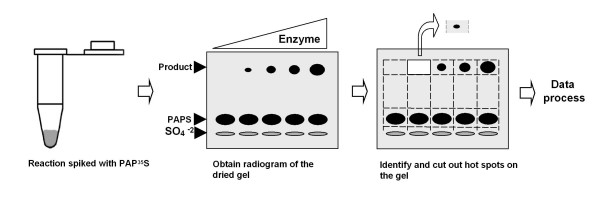
**Scheme of electrophoresis based sulfotransferase assay**. The assay mainly consists of four steps. First, spike sulfotransferase reaction with PAP^35^S. Second, separate the reactions on 8% SDS gel by electrophoresis and obtain an autoradiogram of the dried gel. Generation of the free sulfate is inevitable due to the degradation of PAPS. Third, cut out the hot spots on the dried gel and count the radioactivity. Fourth, data process.

Although the significance and pervasiveness of sulfation have gradually been realized, research on this area has been hampered by the inconvenient assay systems currently available. Usually, sulfotransferase assays rely on chromatography steps, such as HPLC [12-14] and TLC [15-17], to separate substrates and products. However, HPLC is difficult to process multiple samples and TLC is unsuitable to separate proteins and polysaccharides. Recently, HPLC coupled mass spectrometry has been applied to characterize sulfotransferase products [18,19], but it is difficult to assess enzymatic activity due to its nature of being semiquantitative. Colorimetric [20] and fluorescent sulfotransferase assays [21] have also been explored, but these assays are limited to substrates with colorimetric or fluorescent properties. We have developed a versatile electrophoresis-based sulfotransferase assay that can be applied to substrates ranging from small molecules, such as α-naphthol, to large molecules, such as proteoglycans, by taking advantage of the fact that both small and large molecules can be separated from PAPS during SDS-PAGE (Figure 1).

## Results

### Assay development for rhCHST4

**Figure 2:**
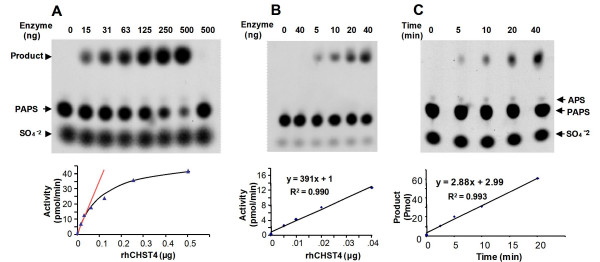
**Assay development for rhCHST4**. The enzyme was assayed with the acceptor substrate of a disaccharide GlcNAcMan. **A) **Activity-enzyme plot and the linear response region. The reactions were performed with fixed substrate inputs (2,500 pmol GlcNAcMan and 1,000 pmol of PAPS) but varied enzyme input from 0 to 500 ng. The calculated activity was then plotted against the enzyme inputs. The linear response region was located from 0 to 40 ng of enzyme input. Last lane contained no acceptor substrate. **B) **The experiment was repeated in the linear region. A linear least-square line was fitted to the activity-enzyme plot and the slope of the line was the specific activity for rhCHST4. The second lane contained no acceptor substrate. **C) **A product-time course of rhCHST4. All reactions contained 7.5 ng enzymes but reaction time varied from 0 to 20 minutes. The relative mobilities of the product and PAPS were slightly different due to variations on the electrophoresis buffer preparation. The faint spots right above the PAPS were likely due to APS, a PAPS degradation product, which was observed especially with aged PAPS preparation.

As an illustration of the general procedure of assay development for all sulfotransferases, the assay for recombinant human carbohydrate sulfotransferase 4 (rhCHST4) is described here (Figure 2). CHST4, also known as high endothelial cell N-acetylglucosamine 6-O-sulfotransferase (HEC-GlcNAc6ST) or L-selectin ligand sulfotransferase (LSST), catalyzes the sulfation at the 6-O position of the non-reducing end N-acetylglucosamine (GlcNAc) residues within mucin-associated glycans [22]. Instead of using native glycan, we used a disaccharide GlcNAcMan as the acceptor substrate.

Each rhCHST4 reaction started with 2,500 pmol acceptor substrate and 1,000 pmol of donor substrate. To locate the linear response region of rhCHST4, an activity-enzyme plot was first established from 0 to 500 ng enzyme input in a 2-fold serial dilution fashion (Figure 2A, Additional file 1, Table S1). The linear response region was then located from 0 to 40 ng of enzyme input and the experiment was repeated within this region. A second activity-enzyme plot was then established and a linear regression equation was obtained based on this second plot (Figure 2B, Additional file 1, Table S2). Because the equation was based on four consecutive data points and had correlation coefficient R^2 ^= 0.990, the slope of the equation, 391 pmol/min/μg, was then accepted as the measured specific activity of rhCHST4.

To make sure that rhCHST4 was stable over the time, a product-time plot was also conducted (Figure 2C, Additional file 1, Table S3). In this experiment, the amount of enzyme was fixed at 7.5 ng, but reaction time varied from 0 to 20 minutes. The enzyme showed a good linear product-time course (correlation coefficient R^2 ^= 0.993), indicating that the enzyme was stable over the time. This result therefore validated the 20 minute reaction time used in previous experiments.

### Assay Examples

**Figure 3:**
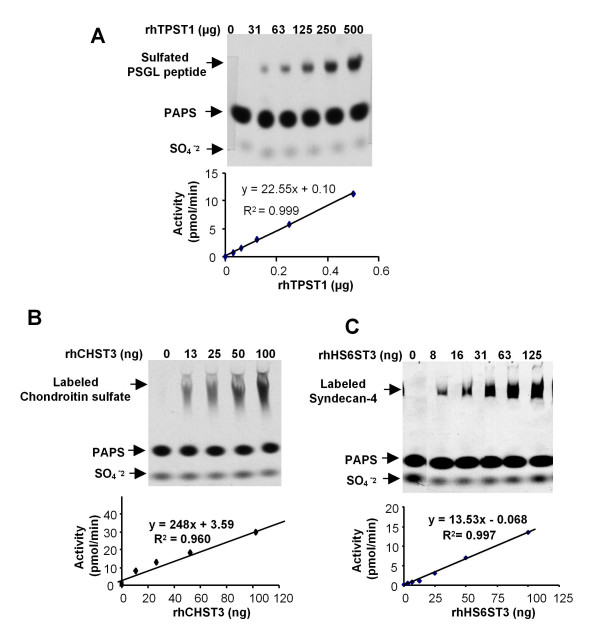
**Examples showing the versatility of the sulfotransferase assay**. **A) **rhTPST1 assay using PSGL-1 peptide as the acceptor. **B) **rhCHST3 assay using chondroitin sulfate as the acceptor. **C)** rhHS6ST3 assay using recombinant human syndecan-4 as the acceptor. See text for experimental conditions.

SDS-PAGE based sulfotransferase assay has been successfully applied to all sulfotransferases that are available to us thus far. To further illustrate the versatility of this method, we describe the assays of several other representative sulfotransferases using different acceptor substrates. To avoid redundancy, only data directly showing specific activity are presented here (Figure 3).

#### TPST1 assay using PSGL-1 peptide as the acceptor

Tyrosylprotein sulfotransferase (TPST) transfers sulfates to the P-selectin glycoprotein ligand-1 (PSGL-1) on myeloid cells and stimulated T lymphocytes and plays a critical role in the tethering of these cells to activated platelets or endothelia that express P-selectin [23]. Recombinant human TPST1 (rhTPST1) was assayed using 25,000 pmol of acceptor substrate PSGL-1 peptide and 1,000 pmol of PAPS in at 37°C for 20 minutes. Enzyme linear response region was located from 0 to 500 ng of enzyme input. Under these conditions, the specific activity was determined to be 22.6 pmol/min/μg (Figure 3A).

#### CHST3 assay using chondroitin sulfate as the acceptor

Carbohydrate sulfotransferases 3 (CHST3), also known as chondroitin 6-sulfotransferase, catalyzes the sulfation at the 6-O position of the N-acetylgalactosamine (GalNAc) residues on chondroitin sulfate [24]. In this example, assay was done using 5,000 pmol of PAPS and 100 μg of bovine chondroitin sulfate as the acceptor substrate. Other assay conditions were the same as those of rhCHST4. Under these conditions, recombinant human CHST3 (rhCHST3) showed a specific activity of 248 pmol/min/μg (Figure 3B).

#### HS6ST3 assay using recombinant human syndecan-4 as the acceptor

Heparan sulfate 6-O sulfotransferase 3 (HS6ST3) transfers sulfate to the 6-O position of GlcNAc residues on heparan sulfate [25]. To assay HS6ST3, recombinant human syndecan 4, a known heparan sulfate proteoglycan, was used as the acceptor substrate. Each reaction was conducted with 10 μg of acceptor substrate and 1,000 pmol of PAPS in the presence of 0.3 mg/mL protamine. Other assay conditions were the same as those of rhCHST4. Under these conditions, recombinant human HS6ST3 (rhHS6ST3) showed a specific activity of 14 pmol/min/μg (Figure 3C). This experiment also proved that the recombinant syndecans 4 carried functional heparan sulfate.

### Relative Mobility of a Small Molecule to PAPS

It is clear that the ability of SDS-PAGE to separate both large molecules, such as recombinant syndecans 4, and small molecules, such as PAPS, is essential to the current assay systems. While it is known that protein separation in SDS-PAGE is mainly due to differences in molecular size, the separation of small molecules during electrophoresis is not well studied. To see whether a small molecule can be separated from PAPS and be applicable to the current assay method, we defined ideal mobility (*M*) as the speed that the small molecule travels in a constant electric field without any resistance. According to this definition, the following equation was derived (Additional file 2),(1)

*q*, charge; *m*, mass; *E*, electric field strength; *t*, running time.

Eq.1 indicates that the mobility of a small molecule under ideal conditions is dependent on electric field strength, running time, and, *q/m *ratio. For all molecules in a same electrophoresis, electric field strength and running time are always the same, but the *q/m *ratios are unique to the molecules. In order to have a more convenient term for sulfotransferase assay, we further defined relative mobility (*M'*) of a small molecule as the ratio of its mobility to that of PAPS (*M*_*p*_).(2)

Since the charge and mass of PAPS (*q*_*p *_and *m*_*p *_respectively) under defined conditions are constant, *M' *of a small molecule is only dependent on its own *q/m *ratio. The value of *M' *suggests whether the molecule can be separated from PAPS during electrophoresis. The estimated relative mobilities of some small molecules encountered in this report are listed in Table 1. Because most of the sulfated small molecules have estimated relative mobilities less than 1, meaning that they move slower than PAPS in an electrophoresis, these molecules are likely to be applicable to SDS-PAGE based sulfotransferase assay.

**Table 1:** Estimated and observed relative mobilities of some of the small molecules that were encountered in this report.

Molecules^a^	Charges^b ^on -SO_3_^-^	Other Charges^b^	*q*	*m*	*q/m *× 1000	Estimated Relative mobility (*M'*)	Observed relative mobility
Dopamine-1S	-1	+1^c^	0	234.2	0.00	0.00	0.05

GlcNAc-Man-1S	-1	0	-1	477.4	-2.09	0.35	0.35

Estradiol-1S	-1	0	-1	352.4	-2.84	0.48	0.37

DHEA-1S	-1	0	-1	367.5	-2.72	0.46	0.45

Lithocholic acid-1S	-1	-1	-2	455.6	-4.39	0.74	0.63

Dopamine-2S	-2	0 ~ +1^d^	-2 ~ -1	312.2/313.2	-3.19 ~ -6.39	0.54 ~ 1.07	0.72

α-Naphthol-1S	-1	0	-1	224.2	-4.46	0.75	0.80

p-nitrophenol-1S	-1	0	-1	218.1	-4.59	0.77	0.96

APS	-1	-1	-2	425.28	-4.70	0.79	0.96

PAPS	-1	-2	-3^e^	504.3	-5.95	1.00	1.00

Bromophenol blue^f^		-2	-2	668	-2.99	0.50	0.63

Free sulfate		-2	-2	96	-20.83	3.5	1.24

^a^Numbers of sulfate groups are indicated as -nS.

^b^The charge numbers on the molecules were estimated based on the numbers of their charged groups, such as -COO^-^, -NH_3_^+ ^and -SO_3_^-^. Because some functional groups were partially charged due to the influence of other neighboring functional groups, the corresponding charge number and relative mobility were only estimation.

^c^Assuming the -NH_3_^+ ^on mono-sulfated dopamine (estimated pKa = 9.5) is completely protonated at pH 8.0.

^d^Assuming the amine group on the di-sulfated dopamine can only partially protonated, because the two negatively charged sulfate groups could shift the pKa of the -NH_3_^+ ^closer to 8.0.

^e ^Fully charged PAPS would have a charge number of -4; however, under the assay conditions, PAPS could be partially charged and the charge number -3 was assumed here. The most similar molecule, ATP, can exist in MgATP^2-^, ATP^4- ^and ATP^3- ^species [34] and overall charge number could be close to -3 too.

^f^Bromophenol blue has about half of the mobility of PAPS and is used as an indicator during electrophoresis.

### Estimated Relative Mobility Correlated Well with Observed Mobility

**Figure 4:**
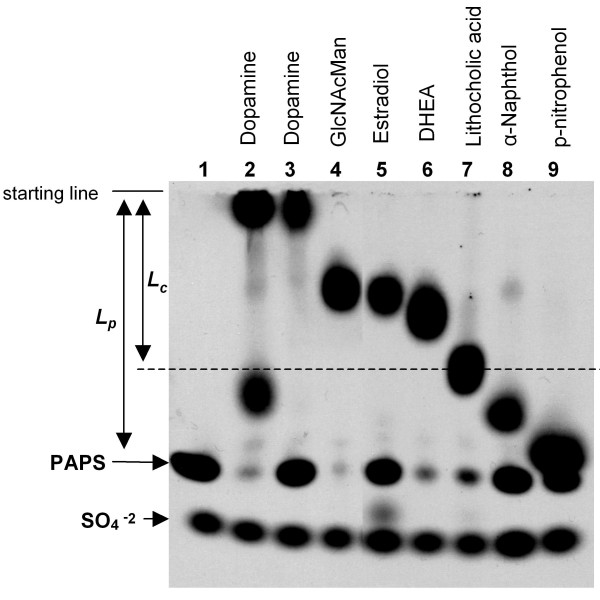
**Relative mobility of various sulfated small molecule products**. To maximize the sensitivity of the detection, no cold PAPS was added into these reactions. Acceptor substrates are indicated on top of the autoradiogram. Lane 1 contained no acceptor substrate but 1 μg of rhSULT1A1. Lane 2, 3, 4 were labeled with 1 μg of rhSULT1A1, rhSULT1E1 and rhCHST4 respectively. All other lanes were labeled with 1 μg rhSULT2A1. Two product bands were observed in rhSULT1A1/dopamine reaction (lane 2) but not in rhSULT1E1/dopamine reaction (lane 3). Acceptor substrate inputs from lane 2 to 9 respectively were: 100 nmol dopamine, 100 nmol dopamine, 20 nmol GlcNAcMan, 10 nmol estradiol, 1 nmol DHEA, 10 nmol lithocholic acid, 10 nmol α-naphthol, and 100 nmol *p*-nitrophenol. The broken line indicates the position of the sulfated lithocholic acid. The distances that sulfated lithocholic acid and PAPS traveled are indicated as *L*_*c *_and *L*_*p*_, respectively. The ratio of *L*_*c *_and *L*_*p*_, 0.63, is the observed relative mobility for mono-sulfated lithocholic acid.

To see how the estimated relative mobilities correlate to the actual relative mobilities for the small molecules listed in Table 1 and further prove that this electrophoresis based assay can be applied to these small molecules, we sulfated corresponding small molecule substrates using SULT1A1, SULT1E1, SULT2A1 and CHST4, and then separated the reactions on an 8% SDS gel (Figure 4). Except the reaction for SULT1A1/dopamine where two products were observed, one product resulted in other reactions.

According to the definition of mobility, the relative mobility of a small molecule should also be equal to the ratio of the distance that it travels (*L*) and the distance that PAPS travels (*L*_*p*_), *i.e.*(3)

The distances of the products and PAPS traveled in Figure 4 were then measured and the observed relative mobilities of these products were obtained (Table 1). The observed values were in the range of ± 0.2 of the estimated values.

The slow and fast moving bands in the SULT1A1/dopamine reaction (lane 2, Figure 4) are likely due to mono- and di-sulfated dopamine respectively, because their observed relative mobilities correlate well with the estimated relative mobilities for mono- and di-sulfated dopamine respectively. While PAPS was almost completely consumed in some reactions (lane 4 and 6, Figure 4), significant amount of PAPS remained in other reactions (lane 3, 5, 8, Figure 4). In each reaction, PAPS was consumed by enzymatic sulfation, the rate of which was determined by enzyme kinetics, as well as degradation, which was evidenced by the presence of free sulfate in each reaction. It was to our advantage to leave some PAPS unconsumed so that we could determine the relative mobilities of the products.

## Discussion

We have developed a sulfotransferase assay in which the separation of sulfated products and donor substrate PAPS is achieved using electrophoresis. In each sulfotransferase assay, an activity-enzyme plot was first obtained to locate the linear response region; a second experiment was then performed within this linear region. The specific activity of the enzyme was the slope of the linear regression equation that was obtained upon the second activity-enzyme plot. Normally, the linear response region was within low percentage of PAPS consumption (<30%) to avoid the buildup of the side products including 3'-phosphoadenosine-5'-phosphate (PAP) and adenosine-5'-phosphosulfate (APS). PAP is reported to be a potent inhibitor to several sulfotransferases [26]. The affect of APS to the assay is less clear and the buildup of APS was only seen in aged PAPS preparation. Because sulfotransferase activities are also highly dependent on substrate concentrations due to normal enzyme kinetics and substrate inhibitions that are unique to some of the sulfotransferases [3,27], all initial substrate inputs were specified in each assay.

This electrophoresis based sulfotransferase assay can be applied to substrates as small as α-naphthol and as large as recombinant proteoglycans. This versatility comes from that SDS-PAGE can separate large proteins and small molecules based on different parameters. Separation of proteins, including proteoglycans, is largely based on size. Separation of small molecules is largely based on *q/m *ratio. For cases where small molecules are used as acceptor substrates, the estimated relative mobility of a sulfated product to PAPS can be used to predict whether the molecule is suitable for electrophoresis-based sulfotransferase assay.

The estimated relative mobility correlated better with the observed values for molecules with defined charge numbers, such as DHEA-1S and GlcNAc-Man-1S, than the molecules with uncertain charge numbers, such as dopamine-2S and lithocholic acid-1S (Table 1). This uncertainty was because of the unknown protonation states of some functional groups that were influenced by neighboring functional groups. A much larger deviation of the estimated relative mobility from the observed relative mobility was observed for free sulfate, which may be due to much stronger counter-ion interaction during electrophoresis.

Although the current method was developed based on assaying various purified recombinant sulfotransferases, it could be applied to assaying enzyme mixture that contains multiple sulfotransferases, as long as the enzymes are specific for different substrates. For example, if an extract contains both HS6ST1 and GalNAc4S6S, we can first assay HS6ST1 activity with heparan sulfate as the acceptor, and then assay GalNAc4S6S activity with chondroitin sulfate A as the acceptor. In general, when a certain type of glycosaminoglycan is used as acceptor, the position of the introduced sulfate is determined by the specificity of the sulfotransferase. However, it is hard to determine the exact sugar residues on a glycosaminoglycan chain that the sulfate is introduced into. To do that, we need more sophisticate methods, such as stable isotope labeling with mass spectrometry analysis [28].

The current electrophoresis-based assay offers several advantages. First, this method allows processing multiple samples simultaneously; therefore it is amenable to inhibitor screening and drug development. Although the current format using standard polyacrylamide gel electrophoresis apparatus is kind of cumbersome, it is possible to miniaturize the systems by taking advantage of scientific advances, such as nanotechnology, and to make the system high-throughput in a similar way that DNA sequencing technology was transformed from polyacrylamide gel electrophoresis based method. Second, it easily separates mono-sulfated and di-sulfated small molecule products, as suggested in the reaction with dopamine and SULT1A1. Although mono-sulfated dopamine with sulfation at either 3-O or 4-O position was reported previously [29], di-sulfated dopamine containing both 3-O and 4-O sulfates has not been reported, probably due to the difficult on separating the two species using traditional methods. The observation of di-sulfated dopamine demonstrates how flexible the substrate binding site of SULT1A1 is [30]. Third, the electrophoresis-based assay is so versatile that it has been successfully applied to all kinds of substrates that have been tested so far. These substrates include proteoglycans, glycosaminoglycans, oligosaccharides and various phenolic small molecules. Finally, because sulfation is biochemically most similar to phosphorylation, it can be reasonably assumed that this method is applicable to kinase assay as well.

## Conclusions

We have demonstrated the versatility of PAGE-based sulfotransferase assay with several representative enzymes, which include carbohydrate and tyrosylprotein specific sulfotransferases. Small molecule specific sulfotransferases such as SULT1A1 can be assayed as long as the sulfated product has different mobility than the donor substrate PAPS. A method for predicting whether a sulfotransferase can be assayed with the current method has also been provided.

## Methods

PAP^35^S was synthesized from carrier free Na_2_^35^SO4 (43 Ci/mg, from American Radiolabeled Chemicals, Inc.), ATP and phosphoenol pyruvate using ATP sulfurylase, inorganic pyrophosphatase, pyruvate kinase and APS kinase, and purified as previously described [31,32]. APS kinase (EC 2.7.1.25) was a gift from Dr. Andrew J. Fisher (University of California-Davis). ATP sulfurylase and inorganic pyrophosphatase were from New England Biolabs. Chondroitin Sulfate, GlcNAcβ1-6Manα1-OMe (GlcNAcMan), heparan sulfate, dopamine, estradiol, DHEA, lithocholic acid, α-naphthol, *p*-nitrophenol, phosphoenol pyruvate and pyruvate kinase were from Sigma-Aldrich. Recombinant syndecan-4 was from R&D Systems. PSGL-1 peptide (QATEYEYLDYDFLPET) was synthesized according to NCBI accession number NP_002997. Cold PAPS was purchased from EMD Biosciences (Cat. No. 118410).

All sulfotransferases were expressed as N-terminal 6× His tagged recombinant proteins. For Golgi resident sulfotransferases, only the luminal enzymatic domains were expressed in CHO cells as secreted forms. For cytosolic sulfotransferases, whole protein sequences were expressed in *E. coli*. The recombinant enzymes were first purified with Ni-affinity resin and then Superdex™ gel filtration or ion-exchange columns (GE Healthcare). Protein concentrations were quantified with Bradford assay [33].

In a typical sulfotransferase reaction, a specified amount of cold PAPS (normally = 1000 pmol), spiked with >10^6 ^CPM of PAP^35^S (< 0.1 picomol), was mixed with specified amounts of acceptor substrate and enzyme in an assay buffer that contained 25 mM MES, 0.5% (w/v) Triton^®^-X 100, 2.5 mM MgCl_2_, 2.5 mM MnCl_2_, 1.25 mM CaCl_2_, and 0.75 mg/ml BSA at pH 7.0 (1× assay buffer) in a final volume of 30 μL. The reaction was then incubated at 37°C for 20 minutes and stopped with 6× SDS stop/loading buffer (100 mM Tris, 10% SDS, 30% glycerol, 60 mM β-mercaptoethanol, and 0.01% bromophenol blue, pH 8.0). The 20-minute reaction time was adopted for all sulfotransferase assays, because most enzymes were found reasonably stable over this period of time at the specified reaction conditions. The reaction was then loaded onto a vertical 8% SDS polyacrylamide gel (Gel buffer: 1.5 M Tris at pH 8.0 with 0.4% SDS). The gel was then subjected to electrophoresis at a constant voltage of 200 volts (~10 v/cm) with a running buffer (50 mM Tris at pH 8.0) for 25 minutes or till the dye (bromophenol blue) was half way to the bottom of the gel. Under these conditions, PAPS would be retained on the gel.

The gel was then transferred onto a cellulose chromatography paper (Fisher Scientific, Cat# 05714-1) and dried in a gel dryer at 80°C under vacuum. The dried gel was tagged with two Glogos^® ^II autorad markers (Stratagene, Catalog # 420202) and exposed to X-ray film for a minimum of two hours. After the film was developed, the positions of the product and PAP^35^S were identified by overlaying the autoradiogram and gel using the markers for alignment. The hot spots were then cut out and counted with a LS 6500 scintillation counter in Ready Safe™ cocktail (Beckman Coulter). The activity of each reaction was calculated with the following equation,(4)

*S *donor substrate input; *C*_*i *_incorporated counts; *C*_*t *_total counts including incorporated and unincorporated; *t *reaction time. Because the actual contribution from PAP^35^S was negligible, the donor input *S *was calculated only based on cold PAPS.

For specific activity determination, an activity-enzyme response plot (activity against enzyme input) was first established based on multiple reactions that covered a wide range of enzyme inputs to locate the linear response region. A second activity-enzyme response plot was then repeated only within this linear response region. The slope of the linear regression equation of this second activity-enzyme plot was then taken as the measured specific activity of the enzyme. For accuracy, the specific activity was considered to be acceptable, only if the equation was based on at least three consecutive points in a two-fold series dilution fashion and had the linear regression coefficient R^2 ^> 0.96.

## Abbreviations

APS: adenosine 5'-phosphosulfate; CHST: carbohydrate sulfotransferase; CPM: counts per minute; DHEA: dehydroepiandrosterone; GlcNAc: N-acetylglucosamine; GlcNAcMan: methyl 6-O-(N-acetyl-β-D-glucosaminyl)-α-D-mannopyranoside; HS6ST: heparan sulfate 6-O sulfotransferase; PAPS: 3'-phosphoadenosine-5'-phosphosulfate; PAP: 3'-phosphoadenosine-5'-phosphate; PSGL-1: P-selectin glycoprotein ligand-1; rh: recombinant human; SDS-PAGE: sodium dodecyl sulfate polyacrylamide gel electrophoresis; SULT: cytosolic sulfotransferase; TPST: tyrosylprotein sulfotransferase.

## Competing interests

The authors declare that they have no competing interests.

The authors are employee of R&D Systems and all researches were founded by R&D Systems. The authors don't have any other financial competing interests.

## Authors' contributions

ZLW conceived the study, designed and performed the experiments and wrote the manuscript. CE performed the experiments and participated in the manuscript writing. SL and BP performed the experiments. WJ participated in the manuscript writing. All authors read and approved the final manuscript.

## Supplementary Material

Additional file 1 **Data calculation for Figure **2. This file contains the raw data and activity calculation for Figure 2. The raw data include the enzyme input and the radio isotope counts for the product and free PAPS in each reaction. Activity calculation was based on the equation *Activity *= *S*·*C*_*i*_/*C*_*t*_·1/*t*, (Eq.4). *C*_*i*_, incorporated counts found in product; *C*_*paps*_, counts of free PAPS; *C*_*t*_, total counts; *S*, donor substrate input; *t*, time. The calculated activities were then plotted out against the enzyme inputs in Figure 2. Click here for file

Additional file 2**Induction of Equation 1**. This file describes how the equation for ideal mobility of a charged small molecule, , was obtained.Click here for file
