# Supplementary material for: A versatile polyacrylamide gel electrophoresis based sulfotransferase assay
Source: BMC Biotechnol. 2010 Feb 10;10:11. doi: 10.1186/1472-6750-10-11 (PMC2834601; doi:10.1186/1472-6750-10-11)
Supplement: Additional file 1 — Data calculation for Figure 2. This file contains the raw data and activity calculation for Figure 2. The raw data include the enzyme input and the radio isotope counts for the product and free PAPS in each reaction. Activity calculation was based on the equation Activity = S·Ci/Ct·1/t, (Eq.4). Ci, incorporated counts found in product; Cpaps, counts of free PAPS; Ct, total counts; S, donor substrate input; t, time. The calculated activities were then plotted out against the enzyme inputs in Figure 2. [file 1472-6750-10-11-S1.DOC]

**Additional file 1, Table S1**, Data calculation for locating rhCHST4 linear response region in Fig. 2A.

| Enzyme  (µg) | C_i_ (cpm) | C_paps_(cpm) | C_t_ =  C_i_ + C_paps_ | C_i_/C_t_ | S (pmol) | P=  S *C_i_/C_t_ | Activity = P/t |
| --- | --- | --- | --- | --- | --- | --- | --- |
| 0.500 | 346406 | 60877 | 407283 | 0.85 | 1000 | 850.53 | 42.5 |
| 0.250 | 244492 | 97001 | 341493 | 0.72 | 1000 | 715.95 | 35.8 |
| 0.125 | 199774 | 214032 | 413806 | 0.48 | 1000 | 482.77 | 24.1 |
| 0.063 | 113555 | 219017 | 332572 | 0.34 | 1000 | 341.44 | 17.1 |
| 0.032 | 86798 | 274897 | 361695 | 0.24 | 1000 | 239.98 | 12.0 |
| 0.016 | 46419 | 290011 | 336430 | 0.14 | 1000 | 137.98 | 6.9 |
| 0.000 | 1404 | 384474 | 385878 | 0.00 | 1000 | 3.64 | 0.2 |

Activity calculation was based on$Activity=S\cdot C_{i}/C_{t}\cdot1/t$, (Eq.4). The calculated activities were then plotted out against the enzyme input in Fig. 2A. C_i_, incorporated counts; C_paps_, counts of free PAPS; C_t_, total counts; S, donor substrate input; P,product; t, time in minutes.

**Additional file 1, Table S2**. Data calculation for repeating rhCHST4 linear response curve in Fig. 2B.

| Enzyme  (µg) | C_i_ | C_paps_ | C_t_ =  C_i_ + C_paps_ | C_i_/C_t_ | S | P=  S *C_i_/C_t_ | Activity = p/t |
| --- | --- | --- | --- | --- | --- | --- | --- |
| 0.040 | 93950 | 196544 | 290494 | 0.32 | 1000 | 323.41 | 16.2 |
| 0.020 | 60025 | 256862 | 316887 | 0.19 | 1000 | 189.42 | 9.5 |
| 0.010 | 35513 | 299225 | 334738 | 0.11 | 1000 | 106.09 | 5.3 |
| 0.005 | 19223 | 284142 | 303365 | 0.06 | 1000 | 63.37 | 3.2 |
| 0.000 | 1245 | 336676 | 337921 | 0.00 | 1000 | 3.68 | 0.2 |

Activity calculation was based on$Activity=S\cdot C_{i}/C_{t}\cdot1/t$, (Eq.4). The calculated activities were then plotted out against the enzyme input in Fig. 2B. C_i_, incorporated counts; C_paps_, counts of free PAPS; C_t_, total counts; S, donor substrate input; P,product; t, time in minutes.

**Additional file 1, Table S3**. Data calculation for rhCHST4 stability test (time course) in Fig. 2C.

| Time  (min) | C_i_ | C_paps_ | C_t_ =  C_i_ + C_paps_ | C_i_/C_t_ | S | P =  S*Ci/Ct |
| --- | --- | --- | --- | --- | --- | --- |
| 0 | 205 | 220840 | 221045 | 0.0009 | 1000 | 0.9 |
| 2.5 | 2131 | 200295 | 202426 | 0.0105 | 1000 | 10.5 |
| 5 | 4023 | 194642 | 198665 | 0.0202 | 1000 | 20.3 |
| 10 | 6176 | 191856 | 198032 | 0.0312 | 1000 | 31.2 |
| 20 | 10156 | 158274 | 168430 | 0.0603 | 1000 | 60.3 |

Activity calculation was based on$Activity=S\cdot C_{i}/C_{t}\cdot1/t$, (Eq.4). The calculated activities were then plotted out against the enzyme input in Fig. 2C. C_i_, incorporated counts; C_paps_, counts of free PAPS; C_t_, total counts; S, donor substrate input; P, product; t, time in minutes.
