# Supplementary material for: A versatile polyacrylamide gel electrophoresis based sulfotransferase assay
Source: BMC Biotechnol. 2010 Feb 10;10:11. doi: 10.1186/1472-6750-10-11 (PMC2834601; doi:10.1186/1472-6750-10-11)
Supplement: Additional file 2 — Induction of Equation 1. This file describes how the equation for ideal mobility of a charged small molecule, , was obtained. [file 1472-6750-10-11-S2.DOC]

**Additional file 2, Induction of Equation 1.**

According to the definition of ideal mobility,

$$M=\frac{L}{t}=\frac{1}{t}\int v\cdot dt$$

According to Newton’s law and electric field theory,

$v=at$, $ma=qE$

*L,* distance that a molecule traveled*; t*, time; *v*, velocity; *a*, acceleration rate; *q*, charge; *E*, electric field strength; *m*, mass.

After combining the above equations,

$$M=\frac{1}{t}\int at\cdot dt=\frac{1}{t}\int\frac{qE}{m}t\cdot dt=\frac{1}{2}Et\cdot\frac{q}{m}$$
